# DeepPIG: deep neural network architecture with pairwise connected layers and stochastic gates using knockoff frameworks for feature selection

**DOI:** 10.1038/s41598-024-66061-6

**Published:** 2024-07-06

**Authors:** Euiyoung Oh, Hyunju Lee

**Affiliations:** 1 Gwangju Institute of Science and Technology, School of Electrical Engineering and Computer Science, Gwangju, 61005 South Korea; 2 Gwangju Institute of Science and Technology, Artificial Intelligence Graduate School, Gwangju, 61005 South Korea

**Keywords:** Tumour biomarkers, Prognostic markers

## Abstract

Selecting relevant feature subsets is essential for machine learning applications. Among the feature selection techniques, the knockoff filter procedure proposes a unique framework that minimizes false discovery rates (FDR). However, employing a deep neural network architecture for a knockoff filter framework requires higher detection power. Using the knockoff filter framework, we present a Deep neural network with PaIrwise connected layers integrated with stochastic Gates (DeepPIG) for the feature selection model. DeepPIG exhibited better detection power in synthetic data than the baseline and recent models such as Deep feature selection using Paired-Input Nonlinear Knockoffs (DeepPINK), Stochastic Gates (STG), and SHapley Additive exPlanations (SHAP) while not violating the preselected FDR level, especially when the signal of the features were weak. The selected features determined by DeepPIG demonstrated superior classification performance compared with the baseline model in real-world data analyses, including the prediction of certain cancer prognosis and classification tasks using microbiome and single-cell datasets. In conclusion, DeepPIG is a robust feature selection approach even when the signals of features are weak. Source code is available at https://github.com/DMCB-GIST/DeepPIG.

## Introduction

Since the era of big data, revolutionary improvements have been made in various fields. A deep neural network (DNN) is a plausible approach for treating complex data. While DNNs offer remarkable predictive abilities in various tasks, their “black box” nature was most concerning to many experts who needed to understand data features used to make such decisions^1,2^. Furthermore, most datasets usually contain features irrelevant to the responses of interest, leading to suboptimal training or overfitting^3,4^. In this context, identifying the crucial features contributing to a specific response and reducing the feature dimensions are essential^5^.

Various feature selection and importance scoring methods have been proposed for statistics and machine learning^6^. Feature selection methods should ideally control the rate of selecting irrelevant features while maintaining high power to identify relevant features. Traditional approaches, including the Benjamini and Hochberg procedures^7–9^, use p-values that reflect feature importance. While these methods are effective for simple models, they face challenges with complex models such as DNNs. In such cases, the generation of meaningful p-values that reflect feature importance becomes ambiguous^10^. Moreover, high-dimensional data incur high costs for computing the p-values.

The model-X knockoff framework for feature selection was proposed to bypass the usage of p-values without violating the false discovery rate (FDR) above a preselected level^11^. The knockoff framework starts by generating knockoff variables that mimic the arbitrary dependence structure among the original features without looking at the responses. Knockoff variables have been used as controls in feature selection by comparing the importance of the original features and their knockoff counterparts.

Recently, feature selection approaches using modified layer architectures from vanilla neural networks have been proposed, such as stochastic gates (STG)^12^. Although they achieved a high detection power in many applications, they often did not consider FDR control explicitly. In addition, most procedures for identifying significant features depend on empirical thresholds, which make them less deterministic. Meanwhile, a DNN architecture suitable for knockoff frameworks, such as DeepPINK, was proposed^13^. DeepPINK introduces a pairwise connected filter layer for the original and knockoff variables. However, it often fails to select a single feature when the signal of important features is dim.

In this study, we designed a novel architecture of DNN and a unique training scheme called pairwise connected layers and stochastic gates (DeepPIG). We combined the core layer architecture of DeepPINK and STG to achieve higher detection power while preventing FDR violations. Furthermore, we developed distinctive training algorithms to compute feature importance using the original and corresponding knockoff variables. From the experimental results, we observed enhanced feature selection performance compared with baseline models using synthetic datasets. DeepPIG exhibited good sensitivity by finding significant features with higher power, especially when the signals of the true features were weak, while ensuring that the FDR was not violated. We also conducted the real data analysis, including cancer survival prediction tasks and the previously used microbiome and single-cell datasets in the baseline model study. The features selected by DeepPIG exhibited better classification performance and robustness than that of DeepPINK. Finally, we report the identified cancer prognostic genes, frequently identified as significant genes for classifying long-term survivors of kidney, liver, and pancreatic cancers. DeepPIG selected several prognostic genes at higher frequencies than the baseline model, highlighting its robustness. Taken together, DeepPIG provides robust feature selection by enhancing selection power and maintaining strict FDR control, making it applicable to various biological datasets.

## Methods

### Knockoff framework

The knockoff filter procedure was introduced as a variable selection method that controls the FDR^11^. The knockoff filter method is well known for providing accurate FDR control while bypassing p-values. The knockoff procedure has two main parts: constructing knockoff variables that imitate the original variables and defining the knockoff statistics that can be taken as feature importance scores. When generating knockoff variables, the responses of interest must not be associated. Formally, knockoff variables for a set of original random variables ${\textbf{x}}=(x_1,...x_d )^T$ are defined as a new set of random variables $\varvec{{\widetilde{\hbox {x}}}}=({\widetilde{x}}_1,...{\widetilde{x}}_d )^T$ that satisfy the following properties:

1. For any subset $S \subset \left\{ 1,...p \right\} ,(x^T,{\widetilde{x}}^T )_{\text {swap}(S)} {\mathop {=}\limits ^{d}} (x ^T,{\widetilde{x}}^T )$, where $(x^T,{\widetilde{x}}^T )_{\text {swap}(S)}$ is obtained by swapping the components $x_j$ and ${\widetilde{x}}_j$ in $(x^T,{\widetilde{x}}^T )$ for each $j\in S$ and ${\mathop {=}\limits ^{d}}$ denotes equal in distribution;
2. $${\widetilde{x}}\perp y\ |\ x$$

Among the methods for constructing knockoff variables, one promising approach is to use DNN-based models such as DeepLINK^14,15^. DeepLINK takes advantage of an autoencoder with flexible nonlinear factor modeling power. Because the feature vectors generated from the autoencoder are nonlinear, one can generate knockoff variables without assuming a joint distribution of *x*, such as Gaussian.

Next, knockoff variables were used as controls for the original variables; therefore, original variables with a significantly stronger relationship with the response than their corresponding knockoffs were considered important features. For each feature index $j=1,...,d$, we defined $K_j$ as the knockoff statistic to measure the importance of the j-th original feature. A large positive value of $K_j$ provides evidence that the jth original feature is important, whereas small magnitudes around zero of $K_j$ are expected to be null features. Formally, knockoff statistics $K_j$ is a function of the augmented data matrix $[{\textbf {X}},\varvec{{\widetilde{\hbox {X}}}}]$ and the response vector y with a function $k_j$ which satisfies the “sign-flip” property:

1 $$\begin{aligned} k_j([{\textbf {X}},\varvec{{\widetilde{\hbox {X}}}}]_{\text {swap}(S)},{\textbf {y}}) = \left\{ \begin{matrix} k_j([{\textbf {X}},\varvec{{\widetilde{\hbox {X}}}}],{\textbf {y}}), j\notin S\\ -k_j([{\textbf {X}},\varvec{{\widetilde{\hbox {X}}}}],{\textbf {y}}), j \in S \end{matrix}\right. \end{aligned}$$

where S denotes any subset of $\left\{ 1,...,d\right\}$. A threshold that does not violate the target FDR is required to select significant features using the constructed knockoff statistics. The set of important features is selected as ${\widehat{S}}=\left\{ j:K_j\ge t \right\}$ with $t=T$ or $t=T_+$, where *T* is the knockoff threshold, and $T_+$ is the knockoff+ threshold. For target FDR level *q*, the knockoff thresholds are defined as follows:

2 $$\begin{aligned} T= & {} \min \left\{ t>0:\frac{\left| \left\{ j:K_j\le -t \right\} \right| }{\max \left\{ \left| j:K_j\ge t \right| ,1\right\} } \le q \right\} \end{aligned}$$

3 $$\begin{aligned} T_+= & {} \min \left\{ t>0:\frac{1+\left| \left\{ j:K_j\le -t \right\} \right| }{\max \left\{ \left| j:K_j\ge t \right| ,1\right\} } \le q \right\} \end{aligned}$$

FDR control was achieved if knockoff statistics of the null features were symmetrically distributed.

### Proposed model

**Figure 1:**
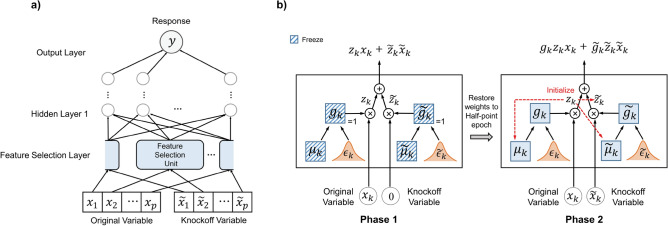
The architecture of DeepPIG and training strategy. (**a**) DeepPIG utilized the feature selection layer incorporating original and knockoff variables to select important features related to responses. (**b**) Detailed view of feature selection unit and their training scheme.

Here, we designed a novel DNN architecture DeepPIG for constructing knockoff statistics to improve the detection power and maintain the FDR control property of knockoff filter methods. We integrated the architectures of STG and DeepPINK^12,13^. The original and knockoff variables were combined and fed into the feature selection unit, followed by the hidden layers (Fig 1a). The final output layer produced the response variable *y*, which is the target output of the network. A detailed view of the feature selection unit and the training strategy are illustrated in Fig. 1b. In the feature selection unit, the stochastic gates are attached to each variable and utilized in a linear combination. STG was employed to utilize the $\ell 0$-norm of features or determine the number of selected features during DNN training. Since exact $\ell 0$ regularization can be computationally expensive and intractable for high dimensions, the stochastic gate component was designed to relax the Bernoulli distribution for the $\ell 0$-norm with a continuous probabilistic distribution. Stochastic gates were attached to each input feature, where the trainable parameter, $\mu _j$, and the random noise, $\epsilon _j$, regulate the probability of the jth gate being active; these are called relaxed Bernoulli variables and are given as follows:

4 $$\begin{aligned} g_j=\text {max}(0,\text {min}(0,\mu _j+\epsilon _j)), \epsilon _j \sim N(0,\sigma ^2) \end{aligned}$$

where *N* denotes the normal distribution with fixed variance $\sigma$. The relaxed Bernoulli variables were clipped, mean-shifted, and random Gaussian. Given a loss *L*, the stochastic gate model is trained by minimizing the empirical risk:

5 $$\begin{aligned} \min _{\theta ,\mu }\mathbb {{\widehat{E}}}_{X,Y}{\mathbb {E}}_{G}[L(f_{\theta }({\textbf{X}}\odot {\textbf{G}},Y)+\lambda \left\| {\textbf{G}} \right\| _{0} )], \end{aligned}$$

where $f_{\theta }$ is a model parameterized by $\theta$, ${\textbf{G}}$ is a random vector with *D* independent variables $g_j$ for $j\in [D]$, *Y* is a response vector and $\odot$ denotes element-wise multiplication. STG considers features to be important if their gate probability values are high, such as 1. Although STG achieved noteworthy performance in finding important features in various experiments, it did not explicitly consider FDR control, which led to the selection of too many features and failure to control FDR in several experimental settings.

Next, we paired the original knockoff variable that successfully passed through the stochastic gate with its corresponding knockoff variable by the plug-in filter layer. The output of this layer is a linear combination of the weighted input variables:

6 $$\begin{aligned} \text {output}_{filter}=g_kz_kx_k + \tilde{g_k}\tilde{z_k}\tilde{x_k} \end{aligned}$$

Through this design, the filter weights connected to the original and knockoff features compete with each other during training. The filter weights corresponding to the original features were much larger if the original features were significant for the response vectors, thereby providing evidence for the selection of important features.

To train DeepPIG, we applied two strategies: (1) a pre-training effect by masking knockoff variables into null vectors in the early stage and (2) a training-stopping criterion using the paired *t*-test results of the knockoff statistics *K*. See Algorithm 1 for the pseudocode of the training scheme. In the first phase, we only fed the original features to the model, replacing the knockoff variables with vectors in which all the elements were set to zero. In addition, gating probabilities $\mu$ and $\widetilde{\mu }$ were frozen with the open state (Algorithm 1 lines 2–5). When the validation loss stabilized, the model weights were restored to a point corresponding to half of the epoch of the stopping point, and the second phase began (Algorithm 1 line 7). For example, if the validation loss stabilized at epoch 10, all model weights were restored to epoch 5. After resetting, the numeric values of the filter weights *z*, connected to the original variables, were copied to the filter weights ${\widetilde{z}}$ which were connected to knockoff variables (Algorithm 1 line 8). Simultaneously, the gating probabilities of both variables ($\mu$ and $\widetilde{\mu }$) were set to 0–1 scaled absolute values of the filter weights of the original variables *z*. This procedure allows the model to roughly identify probable features.

Next, the training was continued with both the original and knockoff variables to drop insignificant features until the model encountered the stopping criterion. The stopping criterion considers the knockoff statistics, which we have revised as follows:

7 $$\begin{aligned} K_j=Z_j-{\widetilde{Z}}_j,j=1,...,d, \end{aligned}$$

where $Z_j=\mu _j (z_j w_j )^2,\ {\widetilde{Z}}_j=\widetilde{\mu }_j ({\widetilde{z}}_j w_j )^2$ and $w\in R^{d}$ are products of fully connected layer weights in the reshaped dimension. Because the importances of $Z_j$ and ${\widetilde{Z}}_j$ are paired and assuming that the distribution of $Z_j$ is greater than that of its counterpart, we applied a paired *t*-test between $Z_j$ and ${\widetilde{Z}}_j$, where the alternative hypothesis is that the mean of the original importance $Z_j$ is greater than that of the knockoff importance ${\widetilde{Z}}_j$. Outliers within two standard deviations of the mean were excluded from this test (Algorithm 1 lines 14–17). This stopping strategy was intended for FDR control because it depends on the assumption that the original and knockoff importance scores of the null features are symmetrically distributed. As the training progressed, the knockoff importance ${\widetilde{Z}}_j$ increased, and the difference between $Z_j$ decreased. The training was stopped when 1) the p-value of the paired t-test was no longer significant $(p > 0.05)$ and 2) the validation loss stabilized (Algorithm 1 lines 18–20). Finally, feature selection was conducted with a knockoff filter procedure using knockoff statistics.

**Algorithm 1:**
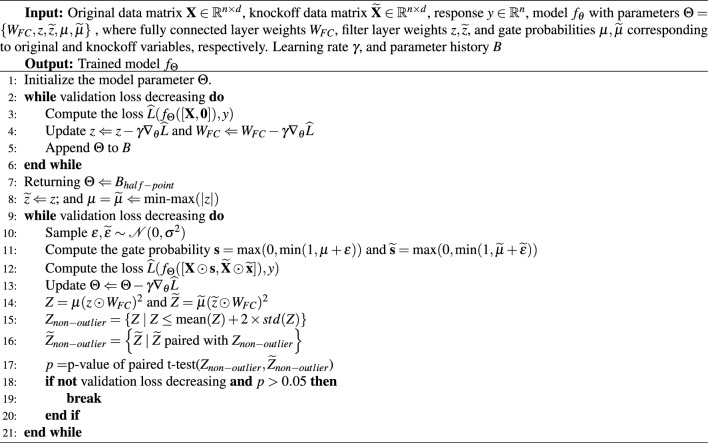
Pseudocode for the DeepPIG training scheme

## Results

### Simulation studies

#### Synthetic data

Mirroring the simulation studies by DeepLINK, we designed the simulation experiment settings as follows: linear factor model and logistic factor model (Eqs. 8, 9).

8 $$\begin{aligned} x_i= & {} \Lambda f_i+\epsilon _i \end{aligned}$$

9 $$\begin{aligned} x_{ij}= & {} \frac{c_j}{1+\exp ([1,{{\textbf {f}}}_i^T] \varvec{\lambda }_j )}+\epsilon _{ij}, j=1,...,d \end{aligned}$$

Here, $f_i=(f_i^1,f_i^2,f_i^3 )^T$ is the latent factor vector, $\Lambda$ and $\varvec{\lambda }_j$ are the factor loading parameters of the desirable dimensions, $c_j$’s are constants, and $\epsilon$ denotes random noise. All the parameters were drawn independently from the standard normal distribution *N*(0, 1). The response vector $y=(y_1,...,y_n)^T$ is assumed to depend on $x_i$ via the following linear and nonlinear link functions (Eqs. 10, 11).

10 $$\begin{aligned} y_\text {linear}= & {} {\textbf {x}}^T\varvec{\beta } \end{aligned}$$

11 $$\begin{aligned} y_\text {nonlinear}= & {} \sin ({\textbf {x}}^T \varvec{\beta })\exp ({\textbf {x}}^T\varvec{\beta }) \end{aligned}$$

where the coefficient vector $\varvec{\beta }=(\beta _1,...,\beta _d )^T$. The locations of the true features were randomly selected, and the corresponding $\beta _j$ was set to the amplitude of nonzero *A*, either positive or negative, with equal probability. The remaining features were considered to be null, and their corresponding $\beta _j$ was set to zero.

Here, we set the sample size *n* to 1000, feature dimension *d* to 500, and true feature size *s* to 10. The values of the amplitude *A* varied from three to 25. For all settings, we conducted experiments 100 times with the target FDR *q* as 0.2.

#### Simulation results

The model’s feature selection performances on the synthetic datasets were determined using metrics such as power and FDR. Power is defined as the expectation of the true discovery proportion (TDP).

12 $$\begin{aligned} \text {Power}:= {\mathbb {E}}[\text {TDP}] \text { with TDP}:= \frac{|S \bigcap S_0|}{|S_0|} \end{aligned}$$

where *S* denotes the subset of selected features and $S_0$ denotes the subset of true features. Power can be interpreted as recall as well. In contrast, FDR is formally defined as the expectation of the false discovery proportion (FDP).

13 $$\begin{aligned} \text {FDR}:= {\mathbb {E}}[\text {FDP}] \text { with FDP}:= \frac{|S \bigcap S_0^c|}{\text {max}\{|S|,1\}} \end{aligned}$$

FDR can be interpreted as 1-precision.

**Figure 2:**
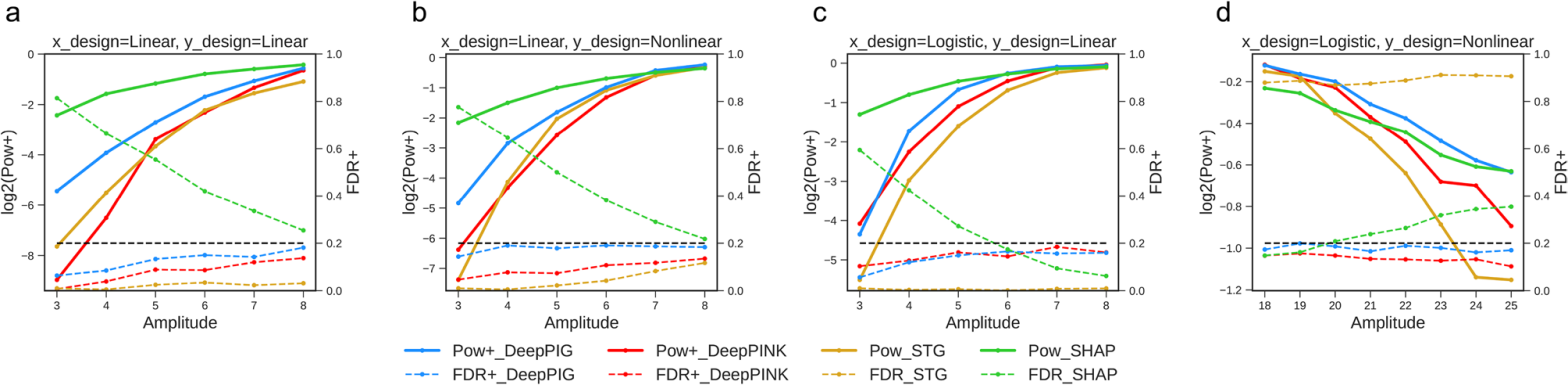
Simulation study results. (**a–d**) DeepPIG, DeepPINK, STG, and SHAP were applied to the synthetic dataset for feature selection. Empirical power and FDR of DeepPIG and DeepPINK were obtained using the knockoff+ threshold. Powers are illustrated as solid lines and FDRs as dashed lines. The preselected FDR target is 0.2, as shown in black dashed lines.

DeepPIG showed better detection power than DeepLINK while controlling the FDR to less than 0.2 in various settings, as illustrated in Fig. 2. When the link function was linear, the signal of the true features increased as the amplitude increased. By contrast, the signal and amplitude are no longer monotonic to the nonlinear link function, as shown in Fig. 2b, d. Notably, DeepPIG showed higher detection power even when the signal amplitudes were weak. Statistically, we conducted the paired *t*-test to compare the obtained powers by DeepPIG and DeepPINK when the amplitudes were three to eight combined. The *p*-values were 1.61 e−08, 1.12 e−14, 5.56 e−08 and 0.695 for the settings described in Fig. 2a–d. For the last case, the *p*-value was 1.20e-08 when amplitudes were 20 to 25 combined. The full scales of the results are depicted in Fig. S1. Further, DeepPIG exhibited higher F1 scores than baseline models when signals were weak, as illustrated in Fig. S2. Based on these observations, it is expected that DeepPIG will be effective in identifying inconspicuous features. STG failed to control the FDR when the response vector was generated using a nonlinear link function, and the amplitudes were large.

We compared the performance of our model with other methods such as SHapley Additive exPlanations (SHAP)^16^, a representative method in the explainable AI area. We utilized the SHAP method on basic DNN models. Furthermore, we applied linear regression and recursive feature elimination (RFE) on Lasso regression for conventional feature selection approaches. We selected the top 10 features based on their shapley values or coefficients, respectively. We observed that these methods were effective in scenarios where the significant features were conspicuous but failed to control FDR when the signals were weak (Fig. 2 and Fig. S3). When the link function was linear, it was difficult to identify important features when the amplitudes were low because the amplitudes and signal were monotonic. SHAP, linear regression, and RFE showed relatively high FDRs when the amplitudes of the features were low. Conversely, when the link function was nonlinear, these methods also exhibited high FDRs for relatively high amplitudes, as the relationship between the amplitudes and signal was not monotonic.

We conducted additional experiments for hyperparameter analysis for the simulation study results. We experimented with the restore epoch that was set to 30% and 90% in addition to 50% to demonstrate the effect of weight transfer timing. The later the weight transfer occurred, DeepPIG tended to select more features. DeepPIG failed to control FDR when the restore epoch was set too late, meaning the knockoff variables were not trained enough. We observed that the optimal performance was achieved when the restore epoch was set to 50% (Fig. S4a).

Next, various ranges of regularization coefficients were assessed to determine their effects on the model parameter. A higher regularization coefficient typically resulted in a controlled FDR with decreased power, leading to selection of fewer features (Fig. S4b). To verify the robustness of our findings, we conducted the experiments again using a synthetic dataset with a different system that had different random seeds and achieved equivalent results (Fig. S5).

### Real data analysis

#### Transcriptomic markers of cancer prognosis

Predicting cancer prognosis is challenging in the field of cancer therapy. In this study, we used DeepPIG to identify cancer prognostic genes. Transcriptomic profiles and prognostic information of patients with kidney, liver, and pancreatic cancer were collected from The Cancer Genome Atlas (TCGA) and International Cancer Genome Consortium (ICGC) databases^17–20^. For each cancer type, we categorized the patients as long-term survivors (LTS) or short-term survivors (non-LTS) based on their survival duration and death event occurrences. Patients were labeled as LTS if their survival times were larger than a specific threshold regardless of their death event occurrence, whereas non-LTS were labeled if their survival times were less than the threshold and the event had occurred. The properties of each dataset are listed in Table 1.

The distance correlation was applied to all three datasets as a screening step before they were fed into the feature selection models^21^. The prediction performance is reportedly poor when these datasets are employed without screening steps, suggesting that the feature space must be reduced before training the models^15^. Using ICGC datasets for screening steps, prognostic genes were ranked by the distance correlation between the gene expression level and LTS status. TCGA datasets were filtered using screened genes and employed in feature selection models.

We applied DeepPIG and DeepPINK 100 times to select the features and compared the number of selected features. For DeepPIG, we set the minimum epoch for weight transfer to increase the sensitivity. For each repetition, we randomly split each dataset into 80% training and 20% testing. To ensure fairness, each repetition used identical knockoff variables and a training-test split for DeepPIG and DeepPINK. Further, repetitions that could not select any features were denoted as “empty repetitions” and excluded from the 100 repetitions when deriving prediction performance. After selection, their prediction abilities were measured using independent vanilla DNNs, and the area under the curve (AUC) and classification errors were determined as the performance metrics. For further analysis, we used all the screened features and the same number of randomly selected features as those selected by DeepPIG for the same repetition. We also computed the precision, recall, and F1 scores and have reported them in Table S7.

We observed that DeepPIG outperformed DeepPINK in terms of the performance metrics and the number of selected features in all three datasets, as summarized in Table 1. Notably, DeepPIG has fewer empty repetitions and greater robustness in feature selection. Despite searching for appropriate hyperparameters, such as the learning rate and regularization coefficients, DeepPINK showed empty repetitions with high chances. The top 10 most frequently selected genes and their selection ratios, i,e., the number of selection times when it was not an empty repetition, are reported in Table 2. DeepPIG showed higher selection ratios for top-ranked genes than DeepPINK, suggesting the robustness of DeepPIG during repetitions. The top 100 genes and their selection ratios are listed in Tables S1–S3.

Finally, we investigated the biological roles and associations of the top-ranked genes with cancers. COL11A1 is essential for bone development and collagen fiber assembly and acts as a prognostic marker in many solid cancers, including renal carcinoma^22–24^. Hepatocyte growth factor (HGF) is a pleiotropic factor that is crucial for tubular repair, regeneration after acute renal injury, renal development, and the maintenance of normal adult kidney structure and function^25,26^. Increased PLOD2 expression is often found in advanced tumors and is correlated with a poor prognosis in patients with hepatocellular carcinoma^27^. It was reported that the overexpression of Stanniocalcin 2 (STC2) was correlated with tumor growth, invasion, metastasis, and prognosis associated with many types of cancers, including liver cancer^28,29^. C15orf48 is highly expressed in pancreatic cancer and is significantly associated with the prognosis of pancreatic adenocarcinoma^30^. Furthermore, the role of RRAD in the occurrence of ferroptosis in pancreatic cancer has been previously reported^31^.

**Table 1:** Long-term survivor classification results for cancer datasets.

Tissue	Dataset$^{\text{a}}$		# of screened features	Mean # of selected features		Empty repetitions		Mean ± SD test AUC (mean ± SD test classification error)$^{\text{b}}$			
	Main	Screening		DeepPIG	DeepPINK	DeepPIG	DeepPINK	DeepPIG	DeepPINK	All screened features	Random features$^{\text{c}}$
Kidney	TCGA-KIRC, 222 LTS (48), 82 nonLTS (24)	RECA-EU, 43 LTS (60) 18 nonLTS (18)	400	9.00	1.92	0 / 100	56 / 100	0.651 ± 0.087 (0.259 ± 0.044)	0.587 ± 0.087 (0.266 ± 0.030)	0.596 ± 0.076 (0.308 ± 0.046)	0.600 ± 0.089 (0.272 ± 0.032)
Liver	TCGA-LIHC, 91 LTS (36), 104 nonLTS (36)	LIRI-JP, 19 LTS (48), 17 nonLTS (12)	200	8.27	2.86	9 / 100	43 / 100	0.631 ± 0.094 (0.404 ± 0.080)	0.596 ± 0.109 (0.419 ± 0.085)	0.661 ± 0.084 (0.374 ± 0.076)	0.570 ± 0.104 (0.455 ± 0.079)
Pancreas	TCGA-PAAD, 66 LTS (18), 66 nonLTS (18)	PAAD-CA, 19 LTS (48), 43 nonLTS (12)	100	5.54	1.26	16 / 100	73 / 100	0.573 ± 0.106 (0.455 ± 0.090)	0.537 ± 0.103 (0.479 ± 0.092)	0.628 ± 0.091 (0.427 ± 0.092)	0.563 ± 0.111 (0.461 ± 0.091)

$^{\rm{a}}$ Dataset name, the number of long-term survivors (LTS) and short-term survivors (nonLTS), and survival time threshold in the month in parentheses.

$^{\rm b}$ Empty repetitions were excluded.

$^{\rm c}$ The same number of features as those of DeepPIG was randomly selected.

**Table 2:** Top 10 frequently selected prognostic genes with selection ratio. (Ratio of times selected to non-empty repetitions).

	Kidney				Liver				Pancreas			
Rank	DeepPIG		DeepPINK		DeepPIG		DeepPINK		DeepPIG		DeepPINK	
1	COL11A1	0.88	IFI44	0.36	PLOD2	0.87	PLOD2	0.33	C15orf48	0.8	C15orf48	0.48
2	HGF	0.88	HGF	0.27	STC2	0.67	ADAM9	0.25	RRAD	0.7	RRAD	0.26
3	IFI44	0.78	COL11A1	0.23	TMX1	0.6	GCLM	0.23	PSMB8	0.52	PSMB8	0.26
4	LYPD6B	0.7	NTM	0.23	ADAM9	0.49	STC2	0.23	GPBAR1	0.45	USP22	0.19
5	KCNE5	0.67	KCNE5	0.18	PARD3	0.41	PIK3IP1	0.19	MAP1LC3B	0.43	RCOR1	0.19
6	BCAT1	0.63	NKAIN4	0.14	IFI6	0.34	MRPL3	0.16	PPP1R10	0.39	FAM19A5	0.19
7	PGC	0.5	LYPD6B	0.14	MERTK	0.31	ZWINT	0.16	TGFBR3	0.27	PPP1R10	0.19
8	C16orf89	0.38	PGC	0.14	GCLM	0.3	PARD3	0.14	UGT2B15	0.19	HIST1H2AC	0.15
9	HSPB7	0.25	BCAT1	0.11	IGFBP3	0.29	GTPBP4	0.12	SH3BP4	0.19	SOX9	0.15
10	APOD	0.24	IRS4	0.11	C5	0.26	MEX3D	0.12	UGT2B17	0.18	GPBAR1	0.11

To further investigate the significance of the selected prognostic genes, we conducted a univariate Cox proportional hazards analysis on the corresponding TCGA cohorts, including all patients whose prognostic information was available. We observed that the top-ranked genes frequently exhibited significant p-values, showing their association with survival in cancer patients, as summarized in Table 3

**Table 3:** Survival analysis of top 10 frequently selected prognostic genes.

Kidney			Liver			Pancreas		
Gene	Hazard ratio	CoxPH p-value	Gene	Hazard ratio	CoxPH p-value	Gene	Hazard ratio	CoxPH p-value
COL11A1	1.018	**0.0001235**	PLOD2	1.027	**5.43E–06**	C15orf48	1.019	**0.000525**
HGF	1.017	**0.00024**	STC2	1.025	**0.0002739**	RRAD	1.007	**0.000857**
IFI44	1.026	**6.39E–12**	TMX1	1.067	**0.0056695**	PSMB8	1.028	**2.94E-05**
LYPD6B	1.017	0.3040778	ADAM9	1.088	**3.83E–05**	GPBAR1	0.919	**0.032268**
KCNE5	1.016	**0.0399017**	PARD3	1.054	**6.63E–05**	MAP1LC3B	1.003	0.865728
BCAT1	1.067	**3.66E–08**	IFI6	1	0.6973015	PPP1R10	0.932	**0.000291**
PGC	1.019	0.0828003	MERTK	1.011	0.214183	TGFBR3	0.994	0.847321
C16orf89	0.997	0.6961147	GCLM	1.017	**1.43E–05**	UGT2B15	1.004	0.323858
HSPB7	0.994	0.7014953	IGFBP3	1.001	0.5672436	SH3BP4	1.016	0.289861
APOD	1.016	**0.0052317**	C5	0.996	**0.0007997**	UGT2B17	0.96	0.351064

p-values under 0.05 are presented in bold.

#### Microbiome and single-cell datasets

We compared the performances of DeepPIG and DeepPINK using the same datasets and procedures described in the DeepLINK study^15^. The datasets used were the human microbiome dataset from a colorectal cancer (CRC) study^32,33^, the human single-cell dataset from a glioblastoma study^34^, and the murine single-cell dataset from a lipopolysaccharide (LPS)-stimulated transcriptomic effect study^35^. Human microbiome datasets were utilized to identify important microbial species related to colorectal cancer by classifying 184 individuals (91 patients with colorectal cancer and 93 healthy controls). The human single-cell dataset contained 632 cells (580 tumor cells and 52 surrounding peripheries) from patients with glioblastoma, and these single-cell gene expression data were employed to investigate the differential gene expression between both cell types. The murine single-cell dataset was collected to investigate the effect of LPS-stimulated nuclear factor-$\kappa$B (NF- $\kappa$B) on gene expression. Classification of 580 cells (202 unstimulated cells and 368 LPS-stimulated cells) based on their condition revealed significantly differentially expressed genes under both conditions.

Similar to the previous section, we applied screening steps and applied DeepPIG and DeepPINK 100 times to select the features. As shown in Table 4, DeepPIG selected more features than DeepPINK and exhibited better test errors. The frequently selected features are listed in Tables S4–S6.

The frequently selected features are similar to those in the analysis in the DeepLINK study. However, some genes were selected more frequently by DeepPIG, suggesting its sensitive detection capability. For instance, for the human microbiome dataset analysis, *Parvimonas micra* was selected 94 times out of 100 repetitions by DeepPIG, whereas it was selected 52 times by DeepPINK. Several recent studies have reported the biological effects of *P. micra* on CRC. *Parvimonas micra* promotes the development of CRC and can be considered as a predictor of poor outcomes in patients with CRC^36,37^. It also influences proliferation, wound healing, and inflammation in CRC cell lines^38^. For human single-cell dataset analysis, B2M and C1R were selected 47 and 42 times, respectively, using DeepPIG, compared to 6 and 3 times using DeepPINK. Some studies have reported that B2M has a significant relationship with the tumor-immune microenvironment and plays a critical role in tumor progression, patient prognosis, and immunotherapy of gliomas^39–41^. Furthermore, the expression level of C1R was associated with immune cell infiltration and prognosis of glioblastoma^42^.

**Table 4:** Performance comparison with microbiome and single-cell datasets.

Dataset	# of screened features	Mean # of selected features		Empty repetitions		Mean ± SD test classification error$^{\text{a}}$			
		DeepPIG	DeepPINK	DeepPIG	DeepPINK	DeepPIG	DeepPINK	All screened features	Random features$^{\text{b}}$
Human microbiome	30	13.69	10.66	0/100	0/100	0.275 ± 0.075	0.287 ± 0.075	0.298 ± 0.072	0.342 ± 0.083
Human single-cell	200	28.93	17.36	0/100	11/100	0.048 ± 0.026	0.053 ± 0.032	0.035 ± 0.022	0.068 ± 0.029
Murine single-cell	200	50.96	34.48	0/100	0/100	0.011 ± 0.013	0.015 ± 0.017	0.009 ± 0.012	0.038 ± 0.031

$^{\text{a}}$ Empty repetitions were excluded.

$^{\text{b}}$ The same number of features as those of DeepPIG was randomly selected.

## Discussion

Various explainable AI (XAI) models such as SHAP^16^ are used for measuring feature importance and understanding which feature contribute significantly to the output of the neural network models. Although the knockoff models and XAI techniques both employ the weights of parameters to compute the feature importance, their main aspects are somewhat different. XAI techniques are mainly applied to interpret the results of trained models that are considered “black box” models that users can not catch how the model comes to the specific results. On the contrary, the knockoff framework is designed to select significant features among a large number of features while keeping the FDR not violated.

One advantage of the knockoff framework over conventional feature selection approaches or SHAP is its ability to determine the threshold using knockoff variables. In other methods, it is necessary to specify the number of features to be selected. We examined the performance of existing methods with the top 10 features, as we were aware that 10 significant features existed within the synthetic datasets. The knockoff framework is useful for real dataset analysis, especially when it is unclear how many features should be selected.

The motivation for designing DeepPIG is that the knockoff filter method often fails to select a single feature in real data analysis. We focused on constructing a sensitive feature-selection model for low-amplitude signal cases. Cancer prognosis prediction is an example of this, as genes related to survival are uncommon. DeepPIG exhibited better detection power when the amplitude of the features was weak, as demonstrated by synthetic and real data.

Some components of the proposed training strategy were determined empirically. For example, in selecting the specific epoch for weight transfer, the late transfer failed in FDR control. If DeepPIG was “overcooked” with the original variable only, the knockoff variables had an insufficient influence on the model and could not overcome the score differences between the original and knockoff variables. In this context, the “half point epoch” criterion was decided empirically based on the simulation study. Although an epoch earlier than half is acceptable, we recommend staying within half of the epoch where the validation loss is stabilized.

Additionally, the 0.05 p-value of the paired *t*-test for the stopping criterion was adjusted. This is because the knockoff statistics within non-outlier regions do not necessarily indicate that they are actual null features. Since a p-value of 0.05 is considered the general criterion in statistical fields, we stuck with it as a criterion for our study. We utilized paired t-tests as a “gadget” to verify how the knockoff importance scores catch up with that of the original. An alternative method for determining the time for weight transfer and testing the symmetry of the original and knockoff scores should be explored in future studies.

## Conclusion

In this study, we present DeepPIG, a DNN architecture, and a training scheme for feature selection. We integrated the key structures of DeepPINK and STG to improve detection power while keeping FDR under a preselected level. Using synthetic data, we achieved a higher power, especially when the amplitudes of the features were weak. We applied DeepPIG to renal carcinoma, hepatocellular carcinoma, and pancreatic carcinoma datasets to classify patients with cancer as LTS or non-LTS. DeepPIG robustly selects several prognostic genes at high frequencies. Furthermore, we compared DeepPIG with the baseline model DeepPINK using the human microbiome, human single-cell, and murine single-cell datasets, which were employed in the baseline model study. It was observed that DeepPIG selected a greater number of features and had superior prediction capacities. In conclusion, DeepPIG is a robust feature selection approach even when the signals of features are weak.

### Supplementary Information

Supplementary Information.

## Data Availability

Simulation data, the human microbiome dataset from a colorectal cancer study, the human single-cell dataset from a glioblastoma study, the murine single-cell dataset from a lipopolysaccharide (LPS)-stimulated transcriptomic effect study, and the cancer prognosis dataset are publicly available at https://github.com/DMCB-GIST/DeepPIG.

## References

[1] Adadi A, Berrada M (2018). Peeking inside the black-box: A survey on explainable artificial intelligence (XAI). IEEE Access.

[2] Saleem R (2022). Explaining deep neural networks: A survey on the global interpretation methods. Neurocomputing.

[3] Roelofs, R. *et al.* A meta-analysis of overfitting in machine learning. *Adv. Neural Inf. Process. Syst.* **32** (2019).

[4] Ying, X. An overview of overfitting and its solutions. *J. Phys. Conf. Ser.* **1168**, 022022 (IOP Publishing, 2019).

[5] Jović, A. *et al.* A review of feature selection methods with applications. In *2015 38th International Convention on Information and Communication Technology, Electronics and Microelectronics (MIPRO)*. 1200–1205 (2015).

[6] Guyon I, Elisseeff A (2003). An introduction to variable and feature selection. J. Mach. Learn. Res..

[7] Benjamini Y (2010). Discovering the false discovery rate. J. R. Stat. Soc. Ser. B Stat. Methodol..

[8] Benjamini Y, Hochberg Y (1995). Controlling the false discovery rate: A practical and powerful approach to multiple testing. J. R. Stat. Soc. Ser. B (Methodol.).

[9] Benjamini, Y. & Yekutieli, D. The control of the false discovery rate in multiple testing under dependency. *Ann. Stat.* 1165–1188 (2001).

[10] Ghorbani A (2019). Interpretation of neural networks is fragile. Proc. AAAI Conf. Artif. Intell..

[11] Candes E (2018). Panning for gold:‘Model-x’ knockoffs for high dimensional controlled variable selection. J. R. Stat. Soc. Ser. B Stat. Methodol..

[12] Yamada, Y. *et al.* Feature selection using stochastic gates. In *International Conference on Machine Learning*. 10648–10659 (PMLR, 2020).

[13] Lu, Y. *et al.* Deeppink: Reproducible feature selection in deep neural networks. *Adv. Neural Inf. Process. Syst.* **31** (2018).

[14] Romano Y (2020). Deep knockoffs. J. Am. Stat. Assoc..

[15] Zhu Z (2021). Deeplink: Deep learning inference using knockoffs with applications to genomics. Proc. Natl. Acad. Sci..

[16] Lundberg, S. M. & Lee, S.-I. A unified approach to interpreting model predictions. In *Proceedings of the 31st International Conference on Neural Information Processing Systems*, NIPS’17. 4768–4777 (Curran Associates Inc., 2017).

[17] Creighton CJ (2013). Comprehensive molecular characterization of clear cell renal cell carcinoma. Nature.

[18] Raphael, B. J. *et al.* Integrated genomic characterization of pancreatic ductal adenocarcinoma. *Cancer cell* **32**, 185–203.e13 (2017). 10.1016/j.ccell.2017.07.007 PMC5964983 28810144

[19] Wheeler DA (2017). Comprehensive and integrative genomic characterization of hepatocellular carcinoma. Cell.

[20] Zhang J (2019). The international cancer genome consortium data portal. Nat. Biotechnol..

[21] Székely GJ, Rizzo ML, Bakirov NK (2007). Measuring and testing dependence by correlation of distances. Ann. Stat..

[22] Li C (2019). Identification of potential core genes in metastatic renal cell carcinoma using bioinformatics analysis. Am. J. Transl. Res..

[23] Wu Y-H, Chou C-Y (2022). Collagen xi alpha 1 chain, a novel therapeutic target for cancer treatment. Front. Oncol..

[24] Nallanthighal S (2021). Collagen type xi alpha 1 (col11a1): A novel biomarker and a key player in cancer. Cancers.

[25] Liu Y (2004). Hepatocyte growth factor in kidney fibrosis: Therapeutic potential and mechanisms of action. Am. J. Physiol.-Renal Physiol..

[26] Liu Y (2002). Hepatocyte growth factor and the kidney. Curr. Opin. Nephrol. Hypertens..

[27] Li K (2023). Dysregulation of plod2 promotes tumor metastasis and invasion in hepatocellular carcinoma. J. Clin. Transl. Hepatol..

[28] Bu Q (2023). Stc2 is a potential biomarker of hepatocellular carcinoma with its expression being upregulated in nrf1α-deficient cells, but downregulated in nrf2-deficient cells. Int. J. Biol. Macromol..

[29] Qie S, Sang N (2022). Stanniocalcin 2 (stc2): A universal tumour biomarker and a potential therapeutical target. J. Exp. Clin. Cancer Res..

[30] Li C (2023). The prognostic and immune significance of c15orf48 in pan-cancer and its relationship with proliferation and apoptosis of thyroid carcinoma. Front. Immunol..

[31] Lu Z (2023). Setd8 inhibits ferroptosis in pancreatic cancer by inhibiting the expression of rrad. Cancer Cell Int..

[32] Yu J (2017). Metagenomic analysis of faecal microbiome as a tool towards targeted non-invasive biomarkers for colorectal cancer. Gut.

[33] Zeller G (2014). Potential of fecal microbiota for early-stage detection of colorectal cancer. Mol. Syst. Biol..

[34] Darmanis S (2017). Single-cell RNA-seq analysis of infiltrating neoplastic cells at the migrating front of human glioblastoma. Cell Rep..

[35] Lane, K. *et al.* Measuring signaling and RNA-seq in the same cell links gene expression to dynamic patterns of nf-$$\kappa$$b activation. *Cell Syst.* **4**, 458–469.e5 (2017). 10.1016/j.cels.2017.03.010 PMC6748049 28396000

[36] Chang Y (2023). *Parvimonas micra* activates the RAS/ERK/c-FOS pathway by upregulating MIR-218-5p to promote colorectal cancer progression. J. Exp. Clin. Cancer Res..

[37] Zhao L (2022). *Parvimonas micra* promotes colorectal tumorigenesis and is associated with prognosis of colorectal cancer patients. Oncogene.

[38] Hatta, M. *et al.**Parvimonas micra* infection enhances proliferation, wound healing, and inflammation of a colorectal cancer cell line. *Biosci. Rep.* **43** (2023). 10.1042/BSR20230609 PMC10272962 37218575

[39] Tang F (2021). Impact of beta-2 microglobulin expression on the survival of glioma patients via modulating the tumor immune microenvironment. CNS Neurosci. Ther..

[40] Zhang H (2021). B2m overexpression correlates with malignancy and immune signatures in human gliomas. Sci. Rep..

[41] Li D (2022). β2-microglobulin maintains glioblastoma stem cells and induces m2-like polarization of tumor-associated macrophages. Cancer Res..

[42] Wang X (2022). C1r, ccl2, and tnfrsf1a genes in coronavirus disease-COVID-19 pathway serve as novel molecular biomarkers of GBM prognosis and immune infiltration. Dis. Markers.

