# Supplementary material for: DeepPIG: deep neural network architecture with pairwise connected layers and stochastic gates using knockoff frameworks for feature selection
Source: Sci Rep. 2024 Jul 6;14:15582. doi: 10.1038/s41598-024-66061-6 (PMC11227546; doi:10.1038/s41598-024-66061-6)
Supplement: Supplementary file 1 — Supplementary Information. [file 41598_2024_66061_MOESM1_ESM.pdf]

DeepPIG: Deep neural network architecture with pairwise connected layers and stochastic gates using knockoff frameworks for feature selection

Euiyoung Oh[1] and Hyunju Lee[1,2,*]

[1]School of Electrical Engineering and Computer Science, Gwangju Institute of Science and Technology, Gwangju, Republic of Korea
[2]Artificial Intelligence Graduate School, Gwangju Institute of Science and Technology, Gwangju, Republic of Korea
[*]Corresponding author. hyunjulee@gist.ac.kr

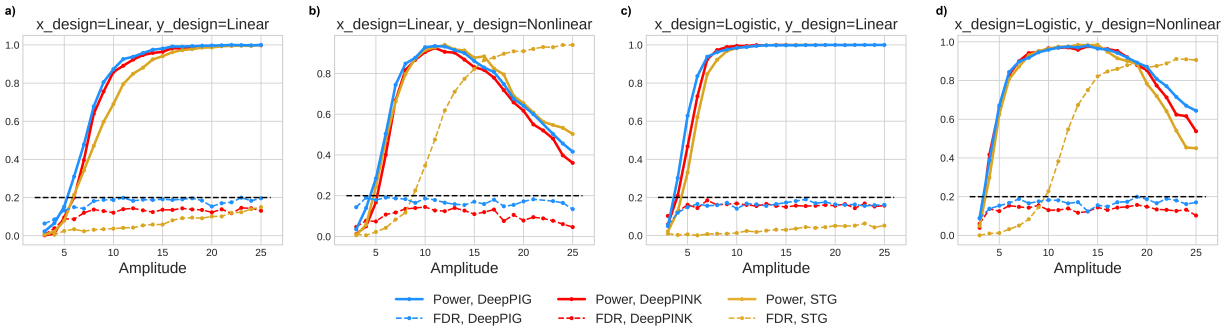

Figure S1: **Simulation study results in a full scale.** (a-d) DeepPIG, DeepPINK, and STG were applied to the synthetic dataset for feature selection. Empirical power and FDR of DeepPIG and DeepPINK were obtained using the knockoff+ threshold. Powers are illustrated in solid lines, and FDR are dashed lines. The preselected FDR target is 0.2, as shown in black dashed lines.

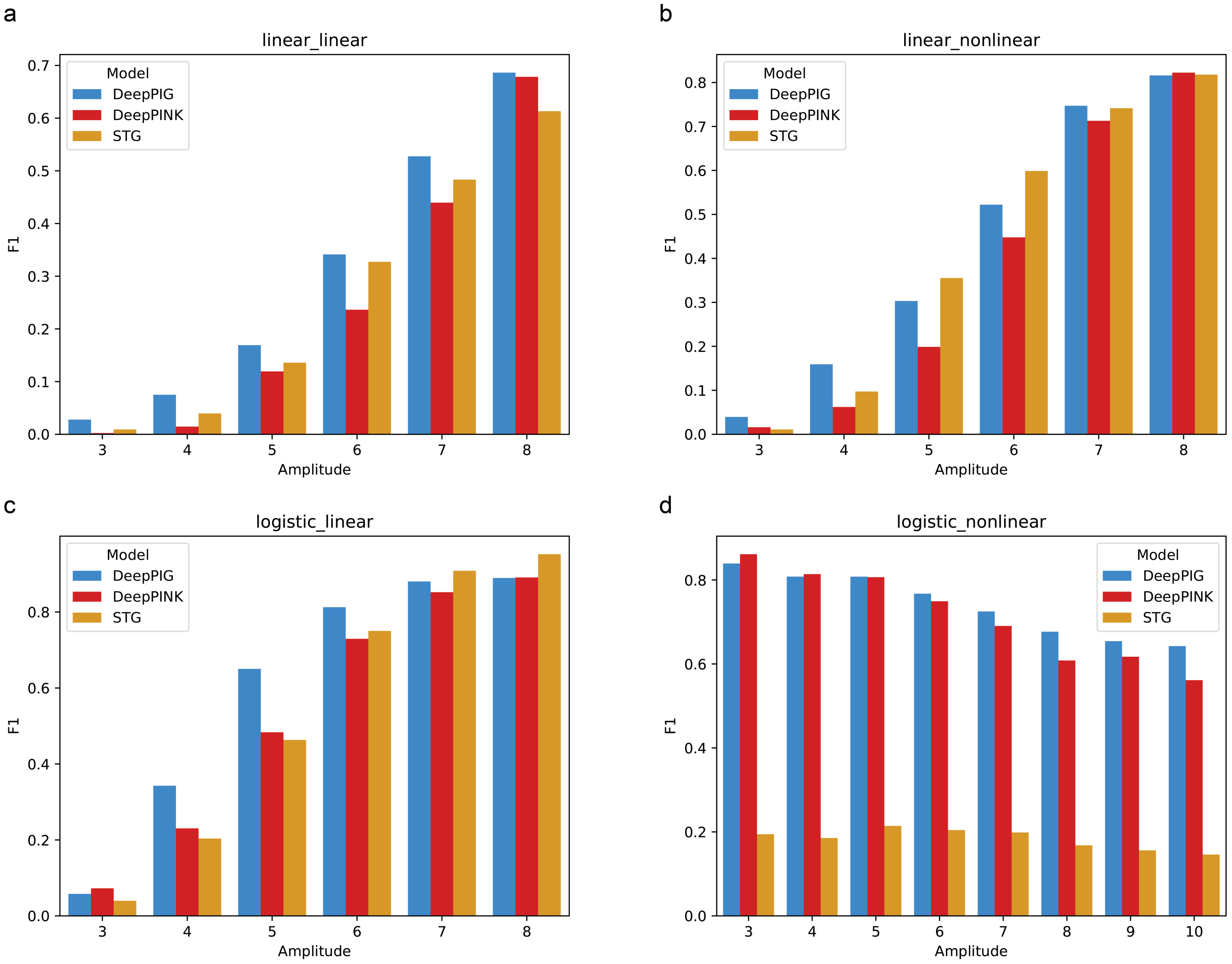

Figure S2: **The average F1 score of the simulation study results** (a-d) DeepPIG, DeepPINK, and STG were applied to the synthetic dataset for feature selection. The average values of 100 repetitions were depicted. DeepPIG showed higher F1 scores when the signals of true features were weak.

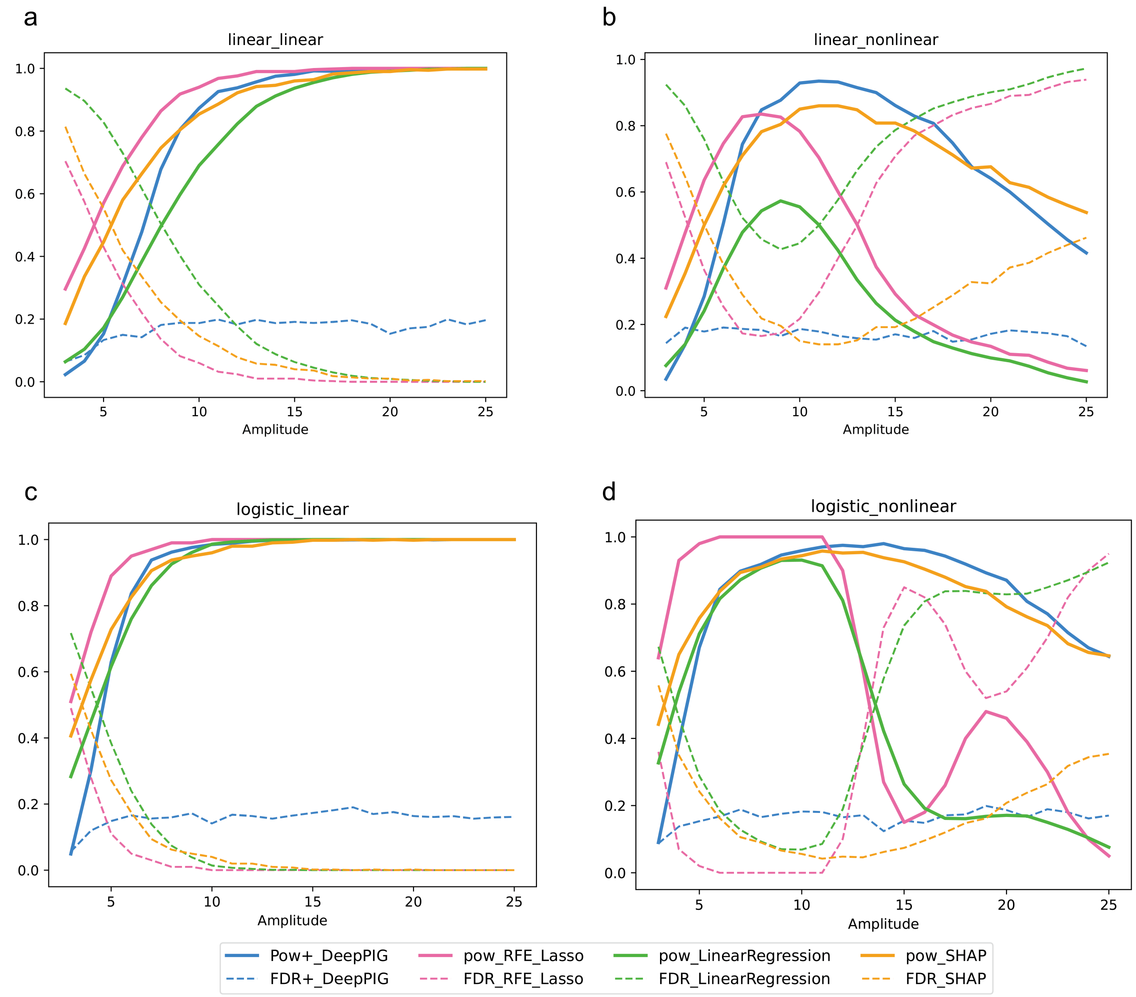

Figure S3: **Feature selection performances of other methods** Linear regression, recursive feature elimination on lasso regression, and SHAP methods were applied to the synthetic datasets. Unlike the knockoff framework, these methods failed to control FDR when the signals of true features were weak.

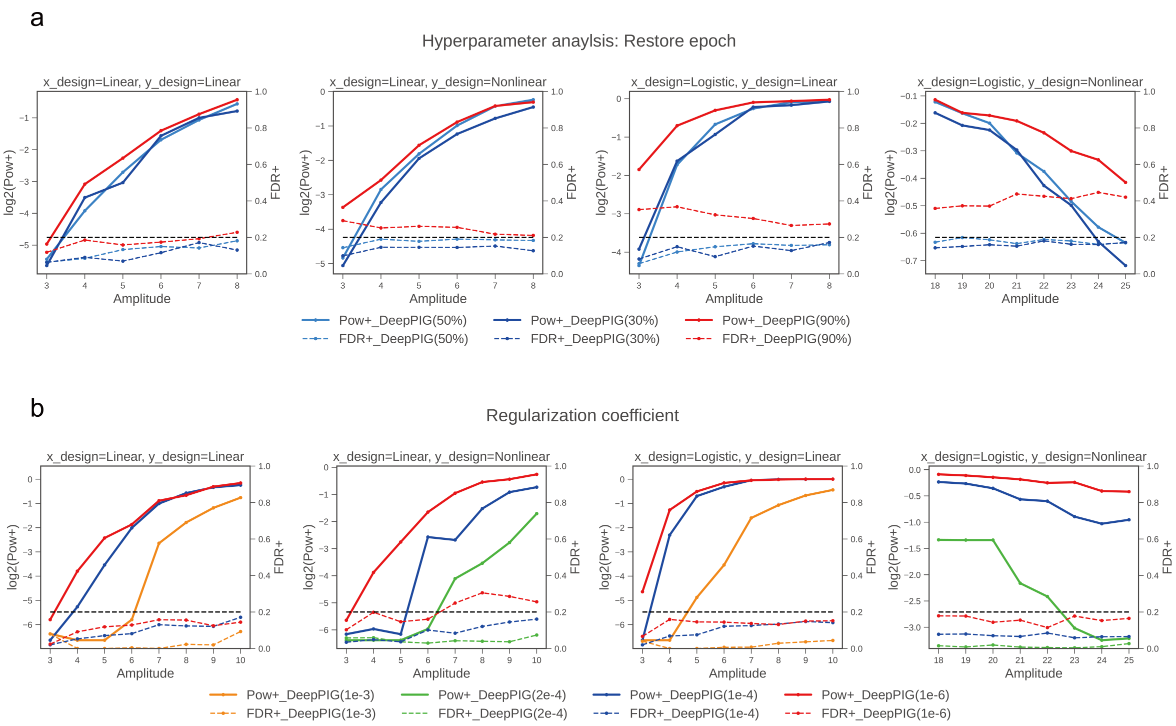

Figure S4: **Hyperparmater analysis** (a) The weight restoration epoch of training phase 1 was varied. When the model was overfitted by original variables only, the knockoff variables got less effect and thus failed to control FDR. (b) The regularization coefficients were varied. The higher values of regularization coefficients tend to select fewer features, decreasing both FDR and power.

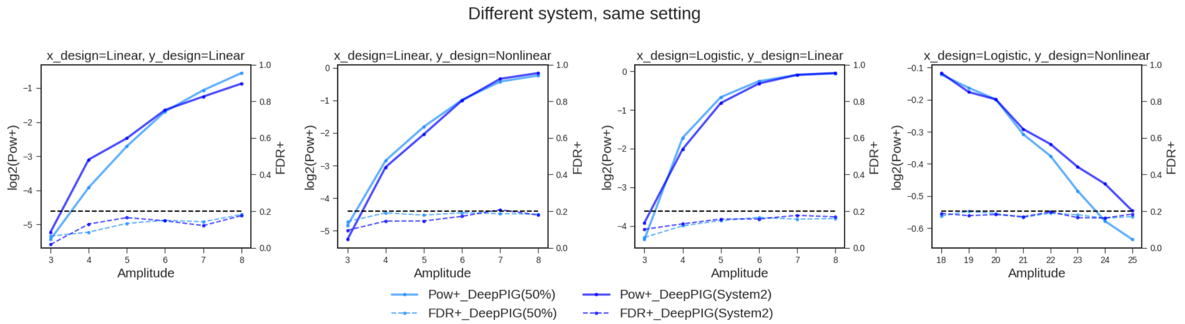

Figure S5: **Replication results on another system** Identical experiments using synthetic datasets were conducted on a different system for replication.

Table S1: Top 100 frequently selected genes in renal adenocarcinoma dataset

Rank	DeepPIG		DeepPINK		Rank	DeepPIG		DeepPINK	
	Gene	Frequency	Gene	Frequency		Gene	Frequency	Gene	Frequency
1	COL11A1	0.88	IFI44	0.36	51	NTM	0.02	BLOC1S1	0.02
2	HGF	0.88	HGF	0.27	52	GPX2	0.02	SMIM5	0.02
3	IFI44	0.78	COL11A1	0.23	53	SPAG7	0.02	MCRS1	0.02
4	LYPD6B	0.7	NTM	0.23	54	TCIM	0.02	FKBP1A	0.02
5	KCNE5	0.67	KCNE5	0.18	55	DKKL1	0.02	AQP6	0.02
6	BCAT1	0.63	NKAIN4	0.14	56	GULP1	0.02	GALNT14	0.02
7	PGC	0.5	LYPD6B	0.14	57	TMEM43	0.02	SLFN5	0.02
8	C16orf89	0.38	PGC	0.14	58	DCXR	0.01	ITPRIPL2	0.02
9	HSPB7	0.25	BCAT1	0.11	59	SERINC3	0.01	CALU	0.02
10	APOD	0.24	IRS4	0.11	60	NR0B2	0.01	RASSF7	0.02
11	DDX42	0.22	MYOF	0.11	61	KNG1	0.01	BSND	0.02
12	SELENBP1	0.21	SELENBP1	0.11	62	PCYT2	0.01	URM1	0.02
13	CEMIP	0.2	CEMIP	0.11	63	TMEM141	0.01	TNFRSF1A	0.02
14	IRS4	0.15	MARS	0.09	64	CBLC	0.01	P3H2	0.02
15	FKBP1A	0.13	DKKL1	0.09	65	PCP4	0.01	TCIM	0.02
16	DGKD	0.13	ADAM33	0.07	66	ITIH5	0.01	FMNL3	0.02
17	PI3	0.12	C16orf89	0.07	67	CETP	0.01	GXYLT2	0.02
18	PHPT1	0.11	KPNA2	0.07	68	HHATL	0.01	MPZL2	0.02
19	ADAM33	0.09	PHPT1	0.05	69	MUC1	0.01	REPS2	0.02
20	DNAJC3	0.09	CALCA	0.05	70	NRP1	0.01	C7orf50	0.02
21	S100A14	0.08	NR0B2	0.05	71	SCGB1D2	0.01	EFR3A	0.02
22	TNFRSF1A	0.08	HHATL	0.05	72	RPP25L	0.01	EXOSC5	0.02
23	GOLT1A	0.07	IGFBP2	0.05	73	MCRS1	0.01	DNAJC3	0.02
24	MPZL2	0.06	WFDC5	0.05	74	SF3B3	0.01	CARD19	0.02
25	SLPI	0.05	UBAC1	0.05	75	NDUFAF8	0.01	SEMA4C	0.02
26	WFDC5	0.05	IFI16	0.05	76	RHOD	0.01	NRDC	0.02
27	F8	0.04	KLK1	0.05	77	HNRNPUL1	0.01	CRYBG1	0.02
28	SFRP2	0.04	GADD45G	0.05	78	ERO1A	0.01	ATP13A2	0.02
29	CFLAR	0.04	GOLT1A	0.05	79	ITPRIPL2	0.01	KHSRP	0.02
30	IL1RL1	0.04	DGKD	0.05	80	P3H2	0.01	SEMA6B	0.02
31	NCSTN	0.04	S100A14	0.02	81	PLOD1	0.01	STT3A	0.02
32	MYOF	0.04	LCN2	0.02	82	C7orf50	0.01	NFE2L3	0.02
33	KPNA2	0.04	HOXB9	0.02	83	EFR3A	0.01	DCXR	0
34	STT3A	0.04	GCGR	0.02	84	EGFR	0.01	STC1	0
35	MARS	0.03	KNG1	0.02	85	TGOLN2	0.01	SSNA1	0
36	LRRN2	0.03	RAB25	0.02	86	RNF145	0.01	MYD88	0
37	ITGB3	0.03	KCNJ1	0.02	87	WDR34	0.01	CLCNKA	0
38	SIRPA	0.03	IGFBP3	0.02	88	FBXO2	0.01	ABCC1	0
39	PDZD2	0.03	OAS2	0.02	89	HSP90B1	0.01	SLPI	0
40	CPE	0.03	LAMA4	0.02	90	HYOU1	0.01	WFDC2	0
41	PALM	0.03	CLEC2B	0.02	91	CRYBG1	0.01	PRDM1	0
42	KLK1	0.03	F8	0.02	92	PPIL3	0.01	SERINC3	0
43	LBR	0.03	SFRP2	0.02	93	EPS8L1	0.01	ARHGAP31	0
44	ADAM15	0.03	CFLAR	0.02	94	DESI1	0.01	ZNFX1	0
45	ABCC1	0.02	DPM2	0.02	95	STC1	0	COQ4	0
46	OAS2	0.02	IL1RL1	0.02	96	SSNA1	0	HAVCR1	0
47	NKAIN4	0.02	ITGB3	0.02	97	MYD88	0	PCYT2	0
48	CX3CR1	0.02	HNRNPM	0.02	98	CLCNKA	0	TRPT1	0
49	IGFBP2	0.02	SLC6A6	0.02	99	LCN2	0	GRB10	0
50	VEGFC	0.02	COX7A1	0.02	100	CALCA	0	MSN	0

Table S2: Top 100 frequently selected genes in hepatocellular carcinoma dataset

Rank	DeepPIG		DeepPINK	
	Gene	Frequency	Gene	Frequency
1	PLOD2	0.87	PLOD2	0.33
2	STC2	0.67	ADAM9	0.25
3	TMX1	0.6	GCLM	0.23
4	ADAM9	0.49	STC2	0.23
5	PARD3	0.41	PIK3IP1	0.19
6	IFI6	0.34	MRPL3	0.16
7	MERTK	0.31	ZWINT	0.16
8	GCLM	0.3	PARD3	0.14
9	IGFBP3	0.29	GTPBP4	0.12
10	C5	0.26	MEX3D	0.12
11	MEX3D	0.25	TMX1	0.12
12	ECHDC1	0.24	CDK1	0.12
13	LYZ	0.2	ENG	0.11
14	HLA-DQA1	0.2	E2F1	0.11
15	CMBL	0.19	MERTK	0.11
16	ZC3H7B	0.14	EIF3M	0.09
17	PIK3IP1	0.14	C5	0.09
18	GTPBP4	0.13	OAS1	0.07
19	ERF	0.13	NUDC	0.07
20	OAS1	0.12	CCT6A	0.07
21	ISM1	0.11	DNAJA2	0.05
22	ERMP1	0.11	IFI6	0.05
23	PZP	0.11	CDCA5	0.05
24	ZWINT	0.1	ELK3	0.05
25	CCT6A	0.1	NCOA5	0.05
26	FLRT3	0.09	ICMT	0.05
27	RNF185	0.09	IL4I1	0.05
28	DHCR7	0.09	RNF185	0.05
29	LYN	0.09	DHCR7	0.05
30	ARHGDIB	0.08	LRIG3	0.05
31	RSAD1	0.08	CIAO1	0.05
32	EIF3M	0.08	KIAA0040	0.05
33	MRPS18B	0.08	ISM1	0.04
34	S1PR2	0.07	RPL29	0.04
35	NCSTN	0.07	ITGB4	0.04
36	HMOX2	0.07	ZC3H7B	0.04
37	E2F1	0.05	PEX11G	0.04
38	NUDC	0.05	DPYS	0.04
39	DPYS	0.04	GRAMD4	0.04
40	MRPL3	0.04	ABCC6	0.04
41	ELK3	0.04	SLC5A6	0.04
42	CCNL2	0.04	TMPRSS6	0.04
43	YIPF5	0.04	ACTN1	0.04
44	SCNM1	0.03	DDX54	0.04
45	FURIN	0.03	UBE2M	0.04
46	NCOA5	0.03	RGS1	0.04
47	SSR4	0.03	CBX1	0.04
48	ABCF3	0.03	ZMIZ1	0.04
49	CDK1	0.03	FCER1G	0.04
50	CANX	0.03	PFKM	0.04
51	GMNN	0.02	ITPA	0.04
52	CHAC1	0.02	NCSTN	0.04
53	MRPL41	0.02	RBBP7	0.04
54	CYP2C8	0.02	CANX	0.04
55	CCNE1	0.02	ME3	0.02
56	MX1	0.02	GMNN	0.02
57	PKN1	0.02	MYBBP1A	0.02
58	RTKN	0.02	PRMT1	0.02
59	PFN1	0.02	IGFBP3	0.02
60	RBP1	0.02	LRRC42	0.02
61	PHLDA1	0.02	TGIF1	0.02
62	LGALS1	0.02	LASP1	0.02
63	FCER1G	0.02	TRIM14	0.02
64	TYROBP	0.02	CHAC1	0.02
65	PFKM	0.02	ATAD3A	0.02
66	EXT1	0.02	DVL3	0.02
67	CIAO1	0.02	GNL3	0.02
68	TCEA1	0.02	ESYT1	0.02
69	ADK	0.02	ECHDC1	0.02
70	DNAJA2	0.01	SNX10	0.02
71	PLIN1	0.01	SLC41A1	0.02
72	MYBBP1A	0.01	RFTN1	0.02
73	PEMT	0.01	ERMP1	0.02
74	SSBP3	0.01	DDX21	0.02
75	LRRC42	0.01	CCNL2	0.02
76	B3GNT2	0.01	ONECUT2	0.02
77	NCOA3	0.01	JAZF1	0.02
78	RPS6KB2	0.01	DEK	0.02
79	TSTD1	0.01	CYTH1	0.02
80	ESYT1	0.01	PHLDA1	0.02
81	GRAMD4	0.01	TBC1D22A	0.02
82	FERMT3	0.01	MAN1B1	0.02
83	ABCC6	0.01	DSG2	0.02
84	C4B	0.01	ABCF3	0.02
85	SLC41A1	0.01	EXT1	0.02
86	SDHC	0.01	TTC1	0.02
87	IL4I1	0.01	PZP	0.02
88	ACTN1	0.01	FBXW11	0.02
89	RGS1	0.01	HLA-DQA1	0.02
90	MRPL10	0.01	YIPF5	0.02
91	ONECUT2	0.01	PDZD11	0.02
92	LRIG3	0.01	ERF	0.02
93	FOLR2	0.01	NIF3L1	0.02
94	CBX1	0.01	HMOX2	0.02
95	TBC1D22A	0.01	TIMM44	0.02
96	ZMIZ1	0.01	ALDH1L1	0.02
97	MAN1B1	0.01	A1CF	0
98	MLLT1	0.01	CMBL	0
99	DSG2	0.01	SLC5A9	0
100	FUBP3	0.01	VIL1	0

Table S3: Top 100 frequently selected genes in pancreatic adenocarcinoma dataset

Rank	DeepPIG		DeepPINK		Rank	DeepPIG		DeepPINK	
	Gene	Frequency	Gene	Frequency		Gene	Frequency	Gene	Frequency
1	C15orf48	0.8	C15orf48	0.48	51	DAG1	0.01	CRB3	0.04
2	RRAD	0.7	RRAD	0.26	52	FAM189B	0.01	IMP4	0
3	PSMB8	0.52	PSMB8	0.26	53	EIF3G	0.01	FGFR4	0
4	GPBAR1	0.45	USP22	0.19	54	NEO1	0.01	P4HA1	0
5	MAP1LC3B	0.43	RCOR1	0.19	55	LSM10	0.01	PPP2R1A	0
6	PPP1R10	0.39	FAM19A5	0.19	56	OSCAR	0.01	BAX	0
7	TGFBR3	0.27	PPP1R10	0.19	57	LGALS2	0.01	NAXE	0
8	UGT2B15	0.19	HIST1H2AC	0.15	58	LAMB3	0.01	YRDC	0
9	SH3BP4	0.19	SOX9	0.15	59	ADH6	0.01	RCC1L	0
10	UGT2B17	0.18	GPBAR1	0.11	60	CENPB	0.01	SEC23A	0
11	VAV1	0.18	MBOAT2	0.11	61	SYNGR1	0.01	MXD4	0
12	ZSWIM7	0.14	FAM189B	0.11	62	ZNF217	0.01	UBE2B	0
13	HNRNPUL1	0.13	CITED4	0.11	63	KLHDC3	0.01	ZC3H7B	0
14	DDAH1	0.12	NEO1	0.11	64	THAP4	0.01	DAG1	0
15	FAM19A5	0.11	UGT2B15	0.11	65	SLK	0.01	TMEM147	0
16	KIAA0100	0.11	AFAP1L2	0.11	66	PEX5	0.01	CYS1	0
17	MBOAT2	0.1	LAMB3	0.11	67	CBX7	0.01	EIF3G	0
18	KCNQ1	0.08	SYNGR1	0.11	68	CRB3	0.01	DNPEP	0
19	DUSP1	0.08	FGD6	0.11	69	FGD6	0.01	CLPP	0
20	DPY19L1	0.07	FNDC3B	0.07	70	RPS11	0.01	CASTOR2	0
21	AFAP1L2	0.07	UGT2B17	0.07	71	ZNF768	0.01	DUSP1	0
22	CLK1	0.07	AHNAK2	0.07	72	FCGBP	0	OSCAR	0
23	PPP2R1A	0.06	PAFAH1B3	0.07	73	IMP4	0	GJB1	0
24	USP22	0.06	SLC25A23	0.07	74	BAX	0	ADM	0
25	MLLT1	0.06	B4GAT1	0.07	75	PAFAH1B3	0	NAGLU	0
26	GJB1	0.06	MLLT1	0.07	76	SEC23A	0	LGALS2	0
27	HIST1H2AC	0.06	KIAA0100	0.07	77	MXD4	0	NDUFAF8	0
28	CAPN6	0.06	SLK	0.07	78	ANGPTL4	0	HS6ST1	0
29	RCC1L	0.05	FCGBP	0.04	79	FAM8A1	0	AQP1	0
30	SLC25A23	0.05	ZSWIM7	0.04	80	ZC3H7B	0	TMSB10	0
31	NAGLU	0.05	MAP1LC3B	0.04	81	TMEM147	0	BHLHE40	0
32	PEPD	0.05	KCNQ1	0.04	82	CYS1	0	HNRNPUL1	0
33	AHNAK2	0.04	ANGPTL4	0.04	83	DNPEP	0	CENPB	0
34	CITED4	0.04	DPY19L1	0.04	84	SLC24A3	0	VAV1	0
35	RCOR1	0.04	FAM8A1	0.04	85	CLPP	0	KIAA1324	0
36	HNF1B	0.04	SOX18	0.04	86	CASTOR2	0	MPC2	0
37	FNDC3B	0.02	SLC24A3	0.04	87	ADM	0	SLC20A1	0
38	YRDC	0.02	GATA6	0.04	88	NDUFAF8	0	ZNF217	0
39	GATA6	0.02	LSM10	0.04	89	AQP1	0	CAPN6	0
40	RNF5	0.02	HNF1B	0.04	90	TMSB10	0	KLHDC3	0
41	HS6ST1	0.02	RNF5	0.04	91	BHLHE40	0	THAP4	0
42	PFKP	0.02	ADH6	0.04	92	KIAA1324	0	FGFR2	0
43	RHOU	0.02	CLK1	0.04	93	MPC2	0	PEX5	0
44	CMTM8	0.02	DDAH1	0.04	94	SLC20A1	0	VEGFA	0
45	FGFR4	0.01	SH3BP4	0.04	95	ABLIM3	0	TBCC	0
46	P4HA1	0.01	PFKP	0.04	96	DBI	0	RHOU	0
47	NAXE	0.01	ABLIM3	0.04	97	FGFR2	0	RPS11	0
48	UBE2B	0.01	DBI	0.04	98	VEGFA	0	ZNF768	0
49	SOX18	0.01	CBX7	0.04	99	SOX9	0	CMTM8	0
50	B4GAT1	0.01	TGFBR3	0.04	100	TBCC	0	PEPD	0

Table S4: Frequently selected features using human microbiome dataset for colorectal cancer

Rank	DeepPIG		DeepPINK	
	Feature name	# of selection	Feature name	# of selection
1	Dialister pneumosintes	100	Dialister pneumosintes	97
2	Eikenella corrodens	99	Eikenella corrodens	89
3	Parvimonas micra	94	Intestinimonas butyriciproducens	79
4	Intestinimonas butyriciproducens	90	Staphylococcus haemolyticus	77
5	Staphylococcus haemolyticus	90	Bacteroides fragilis	60
6	Clostridium bornimense	73	Clostridium bornimense	53
7	Bacteroides fragilis	71	Parvimonas micra	52
8	Latilactobacillus sakei	68	Latilactobacillus sakei	48
9	Clostridium chauvoei	62	Clostridium chauvoei	46
10	Gemella sp. oral taxon 928	61	Gemella sp. oral taxon 928	36
11	Corynebacterium sp. NML98-0116	50	Akkermansia muciniphila	36
12	Ndongobacter massiliensis	45	Streptococcus sp. A12	34
13	Akkermansia muciniphila	45	Corynebacterium sp. NML98-0116	33
14	Streptococcus sp. A12	44	Ndongobacter massiliensis	31
15	Lactococcus garvieae	36	Prevotella intermedia	30
16	Prevotella intermedia	35	Anaerococcus mediterraneensis	30
17	Anaerococcus mediterraneensis	34	Fusobacterium varium	29
18	Escherichia phage HK629	33	Lactococcus garvieae	29
19	Aggregatibacter aphrophilus	30	Ruminococcus bicirculans	26
20	Peptoniphilus sp. ING2-D1G	28	Escherichia phage HK629	24
21	Ezakiella massiliensis	27	Ezakiella massiliensis	22
22	Ruminococcus bicirculans	26	Peptoniphilus sp. ING2-D1G	16
23	Desulfovibrio fairfieldensis	25	Aggregatibacter aphrophilus	16
24	Fusobacterium varium	23	Haemophilus pittmaniae	15
25	Cloacibacillus porcorum	22	Cloacibacillus porcorum	14
26	Haemophilus pittmaniae	17	Streptococcus pyogenes	12
27	Streptococcus pyogenes	16	Clostridium sp. SY8519	10
28	Clostridium sp. SY8519	11	Desulfovibrio fairfieldensis	9
29	Bacteroides cellulosilyticus	7	Faecalibacterium prausnitzii	9
30	Faecalibacterium prausnitzii	7	Bacteroides cellulosilyticus	4

Table S5: Frequently selected features using human single-cell dataset for glioblastoma

Rank	DeepPIG		DeepPINK	
	Gene	# of selection	Gene	# of selection
1	SPARCL1	70	ATP1A2	56
2	QPCT	62	PRODH	43
3	PRODH	60	IGSF21	43
4	SERPINE2	57	HES6	42
5	ATP1A2	54	SPARCL1	33
6	B2M	47	ALDOC	31
7	PSRC1	46	PPM1K	31
8	ALDOC	44	CNTN1	30
9	BAALC	44	SERPINE2	30
10	IGSF21	43	HIF3A	26
11	EFHD1	42	HSPB8	26
12	C1R	42	NPL	25
13	LRRIQ1	37	TMEM98	22
14	ELOB	36	ALDH2	22
15	HSPB8	36	CCND2	22
16	CCND2	33	ACSS1	22
17	HES6	33	PPARGC1A	21
18	PPM1K	31	PLPPR4	21
19	ETNPPL	30	MSMO1	20
20	CFH	29	CFH	19
21	PPARGC1A	29	FGFR3	19
22	SYT9	29	ATP5F1B	19
23	PCSK1N	26	EFHD1	19
24	ALDH2	26	NTRK2	18
25	FGFR3	25	TTPA	17
26	OAZ1	25	BRINP3	17
27	UBXN10	25	LRRIQ1	15
28	TMEM98	23	IFI44L	15
29	GRID2	23	VCAN	14
30	C16orf89	23	QPCT	14
31	NKAIN4	22	ATP1B2	14
32	ATP5F1B	22	LPAR6	14
33	SNX3	22	GRID2	14
34	NPL	22	PARM1	14
35	IFI44L	22	LMO3	13
36	CNTN1	21	NKAIN4	13
37	CPE	21	GLCCI1	12
38	MEGF10	20	ACADM	12
39	ACSS1	20	ASCL1	12
40	PARM1	20	UBXN10	12
41	VCAN	19	SYT9	12
42	BRINP3	19	OAS1	11
43	IDI1	18	CPXM2	11
44	TRAF4	18	SLC14A1	11
45	SCG3	18	SLC18B1	11
46	PLPPR4	18	IDI1	10
47	HIF3A	17	SCG3	10
48	TTPA	17	PSRC1	10
49	OLFML2B	17	SPX	10
50	CPXM2	16	ALDH1L1	10

Table S6: Frequently selected features using murine single-cell dataset for nf-κb expression and lipopolysaccharides response

Rank	DeepPIG		DeepPINK	
	Gene	# of selection	Gene	# of selection
1	Sqstm1	99	Sqstm1	84
2	Hsp90aa1	98	Hspa8	83
3	Sod2	95	Sdc4	78
4	Sdc4	95	Hsp90aa1	76
5	Tnfaip2	94	Sod2	75
6	Hmox1	93	Gmnn	62
7	Hspa8	93	Srsf5	61
8	Chordc1	91	Tnfaip2	61
9	Gadd45b	89	Fdft1	55
10	Srsf5	89	2010111I01Rik	51
11	Gmnn	88	Hmox1	49
12	Ccl3	85	Ly86	46
13	2010111I01Rik	82	Odc1	45
14	Odc1	80	Chordc1	43
15	Fdft1	79	Gadd45b	43
16	Fosb	77	Sulf2	40
17	Txnrd1	77	Hmgcr	40
18	Cyp51	73	Rel	39
19	Cd40	73	Esd	39
20	Zmiz1	71	Dynll1	38
21	Ly86	71	Txnrd1	37
22	Rel	69	Ccl3	36
23	Kdm6b	67	Cyp51	34
24	Slc4a7	67	Zmiz1	34
25	Rhoc	64	Cd40	34
26	Hmgcr	61	Crip1	33
27	Dynll1	60	Cxcl16	33
28	Esd	58	Slc4a7	33
29	Slc3a2	57	Eps8	32
30	Tnip1	56	S100a6	30
31	S100a6	55	Rhoc	30
32	Plek	55	Ifrd1	29
33	Sulf2	54	Polr2e	29
34	Crip1	52	Fcgr1	29
35	Ifrd1	49	Ubc	27
36	Ubc	49	BC005537	27
37	Cxcl16	49	Slc3a2	26
38	Fam162a	45	Lgmn	26
39	Mef2c	44	Fam162a	25
40	Exoc3l4	44	Pik3r5	25
41	Ier3	43	Ier3	24
42	BC005537	43	Mrpl52	24
43	Ubb	40	Kdm6b	24
44	Lgmn	40	Srgn	24
45	Eps8	39	Pgam1	23
46	Mrpl52	38	Plek	23
47	Btg2	37	Tnip1	23
48	Pik3r5	37	Fosb	22
49	Fcgr1	35	Btg2	22
50	Peli1	33	Rrm2	22

Table S7: Precision, recall, and F1 score of long-term survivor classification results for cancer datasets

	Kidney				Liver				Pancreas			
	DeepPIG	DeepPINK	All	Random	DeepPIG	DeepPINK	All	Random	DeepPIG	DeepPINK	All	Random
Precision	.592 ± .205	.432 ± .261	.391 ± .100	.410 ± .270	.647 ± .066	.618 ± .105	.662 ± .077	.561 ± .106	.569 ± .105	.546 ± .210	.550 ± .090	.536 ± .099
Recall	.236 ± .140	.120 ± .144	.361 ± .128	.109 ± .121	.646 ± .157	.579 ± .106	.660 ± .107	.628 ± .176	.559 ± .219	.479 ± .137	.534 ± .122	.563 ± .209
F1	.339 ± .129	.265 ± .150	.369 ± .106	.242 ± .125	.636 ± .076	.592 ± .088	.656 ± .073	.582 ± .110	.560 ± .127	.504 ± .160	.539 ± .100	.542 ± .095
